# Cellular Localization of Exogenous Cry1Ab/c and its Interaction with Plasma Membrane Ca^2+^-ATPase in Transgenic Rice

**DOI:** 10.3389/fbioe.2021.759016

**Published:** 2021-11-02

**Authors:** Jianmei Fu, Yu Shi, Laipan Liu, Biao Liu

**Affiliations:** ^1^ Nanjing Institute of Environmental Sciences, Ministry of Ecology and Environment, Nanjing, China; ^2^ Institute of Plant Protection, Jiangsu Academy of Agricultural Sciences, Nanjing, China; ^3^ College of Life Sciences, Nanjing Agricultural University, Nanjing, China; ^4^ College of Plant Protection, Nanjing Agricultural University, Nanjing, China

**Keywords:** exogenous Cry1Ab/c protein, cellular localization, Ca^2+^-ATPase, protein interactions, transgenic rice, salt resistance

## Abstract

The cellular localization of exogenous proteins expressed in transgenic crops not only determines their stability, but also their effects on crop growth and development, including under stressful conditions; however, the underlying molecular mechanisms remain unknown. Here, we determined the cellular distribution of exogenously expressed Cry1Ab/c protein in insect-resistant transgenic rice Huahui-1 (HH1) cells through subcellular localization, immunohistochemistry, immunofluorescence, and western blot analyses. Interaction between the Cry1Ab/c protein and the preliminarily screened endogenous plasma membrane protein Ca^2+^-ATPase was investigated through yeast two-hybrid, bimolecular fluorescence complementation (BIFC), and co-immunoprecipitation analyses. The potential interaction mechanism was analyzed by comparing the cellular localization and interaction sites between Cry1Ab/c and Ca^2+^-ATPase. Phenotypic indices and Ca^2+^-ATPase activity, which may be regulated by the Cry1Ab/c–Ca^2+^-ATPase interaction, were determined in transgenic HH1 and the parental line Minghui-63 under stress-free and salt-stress conditions. The results showed that Cry1Ab/c was not only distributed in the cytoplasm and nucleus but was also distributed on the plasma membrane, where it interacted with plasma membrane Ca^2+^-ATPase. This interaction partially retain plasma membrane protein Ca^2+^-ATPase in the nucleus by a BIFC experiment and thus may affect Ca^2+^-ATPase activity on the membrane by altering the cellular location of the protein. Consistently, our results confirmed that the presence of Cry1Ab/c in the transgenic HH1 resulted in a reduction in Ca^2+^-ATPase activity as well as causing detrimental effects on plant phenotype, including significantly reduced plant height and biomass, compared to parental MH63; and that these detrimental effects were more pronounced under salt stress conditions, impacting the salt resistance of the transgenic plants. We suggest that the Cry1Ab/c–Ca^2+^-ATPase interaction may explain the plasma membrane localization of Cry1Ab/c, which lacks a signal peptide and a transmembrane domain, and the adverse effects of Cry1Ab/c expression on the growth and development of transgenic HH1 plants under salt stress. This information may clarify the molecular mechanisms of these unintended effects and demonstrate the feasibility of evaluating the success and performance of genetic modification of commercially vital crops.

## Introduction

The *Bt* from *Bacillus thuringiensis* encodes an insecticidal crystal protein that acts specifically against Lepidoptera, Coleoptera, Orthoptera, and Diptera insects, while theoretically being innocuous to humans, animals, and the environment (Betz et al., 2000). Currently, the *Bt* is commonly used for the development of insect-resistant transgenic crops and has been commercialized, generating significant economic, environmental, and social benefits (ISAAA, 2018). Stable, high *Bt* expression is required to achieve the desired insect-resistance effect in the field and therefore is an important factor determining the application prospect of insect-resistant transgenic crops. However, exogenous transgenic expression in plants typically is not stable (Rocher and Green, 1998).

Stable and efficient exogenous protein expression in all tissues and crop growth stages reportedly still cannot be achieved by introducing wild-type *Bt* or even *via* artificial modification, such as the introduction of a strong promoter and enhancer or efficient termination signals, and plant codon optimization (Fischhoff et al., 1987; Vaeck et al., 1987; Perlak et al., 1990; Hoffmann et al., 1992; Sanahuja et al., 2011). Frequently, some target insect larvae can survive by avoiding feeding on tissues and in growth stages with high Bt toxin expression, resulting in crop yield losses and the emergence of resistant insects (Sisterson et al., 2006; Tabashnik and Carriere, 2009; Badran et al., 2016; Xiao and Wu, 2019).

More importantly, the continuous accumulation and expression of exogenous Bt proteins may adversely affect crops’ growth and development. One study reported that productivity was reduced in two highly *cry2Aa*-expressing chickpea lines when compared to that in the parental line and lines with moderate *cry2Aa* expression levels (Acharjee et al., 2010). Similarly, Rawat et al. (2011) found that excessive expression of exogenous Cry1Ac in tobacco and cotton cells was toxic and affected normal crop plant growth and development; however, these adverse effects could be circumvented by targeting the protein to the chloroplasts. Therefore, it would be interesting to explore how Bt protein is transported and stored after it is processed on the ribosomes in transgenic plants.

Saline-alkaline stress is another factor that seriously reduces crop growth and yield. The plasma membrane protein Ca^2+^-ATPase is believed to play key signaling roles in the saline-alkaline stress response (Mansour et al., 1998; Chung et al., 2000; Astegno et al., 2017) and may alter its activity, stability, or subcellular localization. We previously reported that an array of agronomic traits, including tiller number, biomass, and grain weight per plant, were significantly lower in insect-resistant transgenic *cry1Ab/c* rice Huahui-1 (HH1) than those in the parental line Minghui-63 (MH63) under saline-alkaline stress (Fu et al., 2018). Moreover, HH1 showed reduced adaptability to nitrogen deficiency and drought than that its parental line MH63, which may be related to high Bt protein expression in the transgenic line (Jiang et al., 2018a, b). However, the underlying molecular mechanisms remain unknown.

Reportedly, as Bt expression can be increased to more than 5% of the total soluble protein expression without negatively affecting any agronomic crop trait when *cry* genes are introduced into the chloroplast genome (McBride et al., 1995), we hypothesized that unstable exogenous Bt expression and the resulting adverse effects on crop growth and development are possibly directly related to the cellular localization and protein interactions of exogenous Bt protein in transgenic crops. Therefore, in the present study, we aimed to 1) determine the cellular localization of exogenous Cry1Ab/c protein in transgenic rice HH1 through subcellular localization, *in-situ* immunohistochemistry, *in-situ* immunofluorescence, and western blot analyses; 2) preliminarily screen and investigate its potential interaction with plasma membrane Ca^2+^-ATPase through yeast two-hybrid, bimolecular fluorescence complementation (BIFC), and co-immunoprecipitation (Co-IP) analyses, which may contribute to understanding the strong plasma membrane localization of Cry1Ab/c; 3) investigate the Cry1Ab/c–Ca^2+^-ATPase interaction mechanism, which may contribute to Ca^2+^-ATPase activity, by comparing cellular localization and interaction sites between these two proteins; and 4) measure the phenotypic indices and Ca^2+^-ATPase activity of HH1 and MH63, which may assist in elucidating the unintended effects caused by Cry1Ab/c–Ca^2+^-ATPase interaction under unstressful and saline stressful conditions. We expected that our findings could provide a sound theoretical basis for understanding the underlying mechanism of the adverse effects of excessive exogenous expression on the crop plant growth and development under stressful conditions.

## Materials and Methods

### Plant Materials

The rice varieties used in the present study were insect-resistant transgenic Huahui-1 (HH1) containing the exogenous *cry1Ab/c* fusion gene and parental Minghui-63 (MH63) (Tu et al., 1998). Both were obtained from the College of Plant Science and Technology of Huazhong Agricultural University, Hubei, Wuhan, China. HH1 rice and MH63 rice seeds were sterilized with 75% alcohol, washed with clean water, and moved to an incubator at 30°C to avoid light until germination. After the buds grew to 1–2 cm, the seeds were transferred temporarily to a small breeding tray in an incubator with a 16 h:8 h light–dark cycle at 28°C and 80% relative humidity (RH) for cultivation for 20 days (in the three-leaf stage). The HH1 and MH63 rice lines showed substantial differences in plant height (HH1, 16.78 ± 1.26 cm; MH63, 19.12 ± 1.03 cm; *p* < 0.01) and fresh biomass (HH1, 0.07 ± 0.00 g; MH63, 0.11 ± 0.02 g; *p* < 0.01). Then 30 HH1 rice seedlings at equal growth rate were blended into one sample and pulverized in liquid nitrogen to construct the rice cDNA library.

Other remaining rice lines were transplanted to large pots (length, width, and height were 840, 560, and 260 mm), respectively. In the heading stage with the exogenous Cry1Ab/c protein expression at the highest level, fresh flag leaves were fixed with 10% medium formalin for subsequent *in situ* immunohistochemistry and immunofluorescence, other fresh flag leaves were immersed in liquid nitrogen upon collection and were stored at −80°C in an ultralow-temperature freezer for the extraction of cell membrane and cytoplasmic proteins.

*N. benthamiana* was cultivated in a greenhouse under a 16 h:8 h light–dark cycle, daytime temperature of 22–25°C, and nighttime temperature of 18–22°C. After 4–6 weeks (five-leaf stage), the plants were used in the *Agrobacterium* GV3101-mediated transient transformation experiment.

### Gene Cloning and Vector Construction

The RNeasy plant mini kit (Qiagen, Germany) was used to extract and purify RNA from HH1 and MH63 rice leaves, in accordance with the manufacturer’s instructions. One microgram of RNA was used as a template, and cDNA was synthesized from all samples by reverse transcription using the PrimeScript RT Reagent Kit with gDNA Eraser (Takara, Japan). Exogenous *cry1Ab/c* was amplified using HH1 rice cDNA as a template. The Ca^2+^-ATPase were amplified using MH63 rice cDNA as template. The primers are listed in Supplementary Table S1. The gene open reading frame (ORF) sequences were acquired from the NCBI (National Center for Biotechnology Information, Bethesda, MD, United States) database.

The recombinant plasmid used in present study are listed in Supplementary Table S1. In-Fusion HD Cloning Kits (Takara, Japan) reaction system was established to homologously recombine Ca^2+^-ATPase into subcellular localization vector pBINPLUS-GFP, bimolecular fluorescence complementation (BIFC) vector PCV-cYFP-N (nYFP) and yeast two-hybrid vector pGBKT7 (BD) by using BamHI single digestion site. The exogenous gene *cry1Ab/c* was cloned into subcellular location vector pBINPLUS-mCherry, BIFC vector PCV-nYFP-C (cYFP) and yeast two hybrid vector pGADT7 (AD).

### Protoplast Isolation and Transfection of Rice

Protoplast isolation and transfection of rice were performed according to Zhang et al. (2011). Protoplasts of approximately size 7–25 µm were generated. After incubation for 16–20 h in 25°C, protoplasts were observed using a Zeiss LSM750 confocal laser-scanning microscope (Carl Zeiss AG, Oberkochen, Germany). GFP were excited at 488 nm wavelengths. The fluorescence experiments were performed three times with randomly selected six protoplasts fields of microscope in per replicate.

### Transient Expression System Developed by *Agrobacterium*-Mediated Transformation

A transient expression system was developed by Agrobacterium-mediated transformation as previously described (Zhang et al., 2011). Before observing the fluorescence, the tobacco leaves were excised and mounted on slides in water. Green fluorescence GFP, red fluorescence mCherry and yellow fluorescence YFP were observed by a Zeiss LSM750 confocal laser-scanning microscope.

### Subcellular Localization

The target genes infused to the pBINPLUS-GFP and pBINPLUS-mCherry vectors were transformed into Agrobacterium strain GV3101 to infiltrate *N. benthamiana* leaves (Zhang et al., 2011). After 48 h, the GFP and mCherry fluorescence signals were read under a Zeiss LSM750 confocal laser-scanning microscope.

### *In-Situ* Immunohistochemistry and Immunofluorescence

For *in-situ* immunohistochemistry, the fresh HH1 and MH63 rice leaves were fixed with 10% formalin neutral fixative solution containing formaldehyde, phosphate and deionized water (Solarbio, China) for more than 24 h, and then the leaves were dehydrated with a graded series of ethanol solutions. The tissues were embedded in paraffin blocks, and cut into 4-µm-thick sections using a Leica cryostat microtome CM 1 950 (Leica, Germany) and mount the sections on slides. Then according to the manufacturer’s instructions of “Immunohistochemistry Protocol (Paraffin) for Application Testing (ThermoFisher, American)” with some modifications, to bake the paraffin at 60°C in a dry oven, remove the paraffin and rehydrate tissue using xylene and a graded series of ethanol (xylene I for 10 min, xylene II for 10 min, anhydrous ethanol I for 5 min, anhydrous ethanol II for 5 min, 95% alcohol for 5 min, 90% alcohol for 5 min, 80% alcohol for 5 min, 70% alcohol for 5 min, and distilled water for 10 min). Using blocking reagent (0.3% hydrogen peroxide methanol solution) to cover the tissue, and using antigen retrieval solution [EDTA buffer (pH 8.0)] by boiling (at 95–100°C) in a microwave oven maintained for 20 min to repair antigen. After washing it with 1×PBS buffer (PH 7.4) three times, tissues were hybridized with Cry1Ab primary antibody (1:50) and the sheep anti-rabbit IgG secondary antibody (1:500). Finally, using Mayer’s hematoxylin to counterstain nucleus. Sealing the edge of the coverslip and drying for 1–2 h before viewing on a microscope (Leica DM IL LED FLUO, Germany).

For *in-situ* immunofluorescence, the methods of paraffin sectioning, antigen repair, and Cry1Ab primary antibody incubation were similar as described above. The slices were incubated with the secondary antibody (1:500), placed in 1×PBS buffer (pH 7.4), and washed three times on a Qilinbeier decolorizing shaker KB-800 at 50 rpm (Qilinbeier, China) for 3 min per wash. After drying the slices, sheep anti-rabbit IgG fluorescent secondary antibody (1:500, ThermoFisher, America) was added into the circle and incubated at room temperature for 3 h. Sealing the edge of the coverslip and drying for 1–2 h before viewing on a microscope (Leica DM IL LED FLUO, Germany).

### Detection of the Cry1Ab/c Protein in Cell Cytoplasm and Membrane by Western Blotting

The cell membrane and cytoplasmic proteins in rice leaves were extracted using a cell membrane cytoplasmic protein extraction kit (Biyuntian, China). The BCA protein quantitation kit (Beyotime, China) was used to quantify rice leaf membrane and cytoplasmic protein samples. Protein extracts (20 ug) were isolated using 4–20% prefabricated sodium dodecyl sulfate–polyacrylamide gel electrophoresis (SDS-PAGE) gels (Bio-Rad Laboratories, Hercules, CA, United States) in 1× running buffer (25 mM Tris, 192 mM glycine, 0.1% SDS) according to Mini-PROTEAN Precast Protein Gels instruction manual and application guide. Stopping the run when the dye front reaches the reference line imprinted on the bottoms of the cassettes, the proteins were transferred onto PVDF membrane using a Tris–glycine membrane transfer system (40°C, 40 V, 14–18 h). With the rabbit antibody of beta-actin (Abcam, Cambridge, United Kingdom) (1:1500) as the internal standard, the plasma membrane H^+^-ATPase and target Cry1Ab/c protein was detected using mouse antibody of Cry1Ab (Abcam, Cambridge, United Kingdom) (1:1500) and rabbit antibody of H^+^-ATPase (Agrisera, Sweden), respectively. The samples were incubated with goat anti mouse IgG (Abcam, Cambridge, United Kingdom) (1:2000), goat anti rabbit IgG (Abcam, Cambridge, United Kingdom) (1:2000), and marker antibodies (Bio-Rad) at the same time, and then with the electrochemiluminescence (ECL) containing 100 ml Clarity Western Peroxide Reagent and 100 ml Clarity Western Luminol/Enhancer Reagent after capturing images (six images were captured in per replicate). Finally, the PVDF membrane was incubated with ECL color developer and enhancement solution (1:1 mixture) for 5 min. The color bands on the membrane were detected using the VersaDoc imaging system (Bio-Rad).

### Yeast Two-Hybrid Screening

The yeast two-hybrid preliminary screening were conducted according to the instructions for the Matchmaker Gold yeast two-hybrid system (Clontech, Japan). For large-scale extraction and identification of endogenous rice proteins interacting with exogenous Cry1Ab/c protein, the aforementioned cDNA library was constructed to the prey vector pGADT7 (AD) and the *cry1Ab/c* gene from HH1 cDNA template amplification was constructed to the bait vector pGBKT7 (BD). The vectors harboring the cDNA library and the bait gene were transformed into the yeast cells Y2H Gold and the *Saccharomyces cerevisiae* strain Y187 (as a mating partner for the Y2HGold reporter strainis for use with Clontech’s Matchmaker Gold Yeast Two-Hybrid System) (Clontech, Japan), respectively. Double-deficiency SD-Trp-Leu (DDO) and quadruple -deficiency SD-Trp-Leu-His-Ade-X-a-gal (QDO/X-a-gal) screening assays were run on them following a previous study (Ni et al., 2019). Positive colonies on the quadruple-deficiency screening plates were selected as the templates and PCR amplification and sequencing were conducted to detect the inserted fragments. The 15 positive clones with the size ranged between 600 and 2000 bp then were checked to confirm the presence of “in frame” cDNA-AD fusion. Some clones (Ca^2+^-ATPase and V-H^+^-ATPase) were found in full-length of cDNA, but the other clones were not. NCBI (National Center for Biotechnology Information, Bethesda, MD, United States) was consulted to name the gene.

### Yeast Two-Hybrid Assay

The yeast two-hybrid point-to-point verification assay was carried out using the Matchmaker Gold Yeast Two-Hybrid System (Clontech, Japan). The target genes fused to the AD and BD vectors were transformed into yeast competent cells Y2H Gold. Each transformation was carried out with 200 µl of 0.9% NaCl suspension yeast, which was as mild as possible. The co-transformed yeast cells were coated on a double-deficiency medium of DDO, and then transferred onto a deficiency medium of QDO/X-a-gal and allowed to grow for 4 days prior to observation.

### Bimolecular Fluorescence Complementation Assay

The target genes infused to the nYFP and cYFP vectors were transformed into Agrobacterium strain GV3101 to infiltrate *N. benthamiana* leaves (Zhang et al., 2011). The 4A-nYFP (NbeIF4A) + P2-cYFP (RSV-encoded proteins) was used as a positive control (Chen, 2015). After 48 h, the YFP fluorescence signals (514 nm) were read under a Zeiss LSM750 confocal laser-scanning microscope (Carl Zeiss AG, Oberkochen, Germany).

### Co-Immunoprecipitation Assay

The target genes infused to the pBINPLUS-GFP and pBINPLUS-mCherry vectors were transformed into *Agrobacterium* strain GV3101 to infiltrate *N. benthamiana* leaves by *Agrobacterium*-mediated transformation as described previously (Zhang et al., 2011). At 48 hpi, leaf samples were harvested and frozen in liquid nitrogen. 1.0 g infiltrated tobacco leaves were pulverized with liquid nitrogen in a mortar. Thereafter, 2 ml of extraction buffer (20 mM DTT, 1× proteinase, 50 mM Tris, 150 mM NaCl, 0.5 mM EDTA, 8% glycerin) was added and the homogenate was milled for 2 min. To the resulting homogenate, we added 50 µl of 20% (v/v) Triton [final concentration 0.5% (v/v)] and the macerate was ground for 1 min. The slurry was then transferred to a 2-ml Eppendorf tube. All samples were vortexed, mixed, and placed in a 4°C refrigerator, mixed by repeated inversions in a rolling-over instrument (Qilinbeier, Suzhou, China), and incubated for 20 min. Then the protein extracts were centrifuged for 15 min at 15,200 g at 4°C. According to the manufacturer’s instructions of GFP-Trap^®^ (Chromotek, Germany), the GFP-Trap agarose beads were used to immunize the protein supernatants, and then were washed by using IP buffer (20%Triton 50 ul, 1M DTT 10 ul and extraction buffer 9.4 ml for per sample). Finally, the agarose beads were diluted with 5× SDS buffer and boiled at 95°C for 10 min. After centrifugation, the supernatant proteins were isolated using 4–20% prefabricated SDS-PAGE (Bio-Rad Laboratories, Hercules, CA, United States) followed by western blot with an anti-GFP antibody (1:2,000) and anti-mCherry (1:1,500) (Abcam, Cambridge, United Kingdom).

### Salt Stress Treatment of Rice Plants

After the vermiculite and nutrient soil (in 0.2–0.5 g/cm^3^ dry weight soil, containning 3.0–5.0% total nitrogen, total phosphorus, total potassium contents; 6.0–10.0% available nitrogen, phosphorus, potassium contents; ≥35% organic matter and 5.8–7.0 pH) was mixed in the proportion of 1:1, 400 rice seedlings of HH1 and MH63 above-mentioned were planted and grew for about 1 week. Our dose-response analysis determined that a final concentration of 150 mM NaCl was used in the salt stress treatment of rice plants (data not shown). After using 150 mM NaCl to treat these rice plants for 9 days, 20 individuals per treatment were randomly chosen to measure plant height and dry biomass. Other remaining samples (whole plant) were collected and preserved at −80°C in an ultralow-temperature freezer for isolating plasma membrane vesicles using the aqueous two-phase separation method (Lin and Morales, 1977; Mansour et al., 1998; Yang et al., 2010; Xue et al., 2018), including five biological replicates. Briefly, plants were homogenized with an isolation buffer [containing 0.33 M sucrose, 10% (w/v) glycerol, 0.2% (w/v) BSA, 5 mM EDTA, 5 mM DTT, 5 mM ascorbate, 0.2% (w/v) casein, 0.6% (w/v) polyvinylpyrrolidone, 1 mM PMSF, 13 protease inhibitor, and 50 mM HEPES-KOH, pH 7.5], and centrifuged at 10000 g for 10 min. The supernatant was filtered through two layers of Miracloth, and centrifuged at 100000 g for 1 h. The pellet was homogenized, resuspended and standed for a few minutes until phase-segregated. Then the upper phase was collected and centrifuged at 100000 g for 1 h. The pellet was ground and resuspended for PM Ca^+^-ATPase activity analysis. All steps of the procedure were carried out at 0–4°C. The protein concentration of plasma membrane vesicles was determined using Bradford method (Beyotime, China). The 50 μg plasma membrane protein were prepared for further to detect the activity of Ca^2+^-ATPase using inorganic phosphate in the enzymatic resolution reaction (Lin and Morales, 1977). Briefly, the measurement were carried out at 30°C for 30 min in 0.5 ml reaction volume containing 1 mM Tris-ATP, 2 mM CaSO_4_, 40 mM Tris-MES, and 0.02% triton X-100, with or without 25 mM K_2_SO_4_. The reaction was started by addition of the enzyme and terminated by adding 1 ml of color development solution [containing 0.75% (w/v) (NH_4_)_6_Mo_7_O_24_, 0.375M H_2_SO_4_, 3% (w/v) FeSO_4_ and 0.75 (w/v) SDS]. Inorganic phosphate was determined spectrophotometrically at 660 nm. The control was un-stressful growth conditions.

## Results

### Cellular Distribution of Exogenous Cry1Ab/c Protein in Plant Cells

**FIGURE 1:**
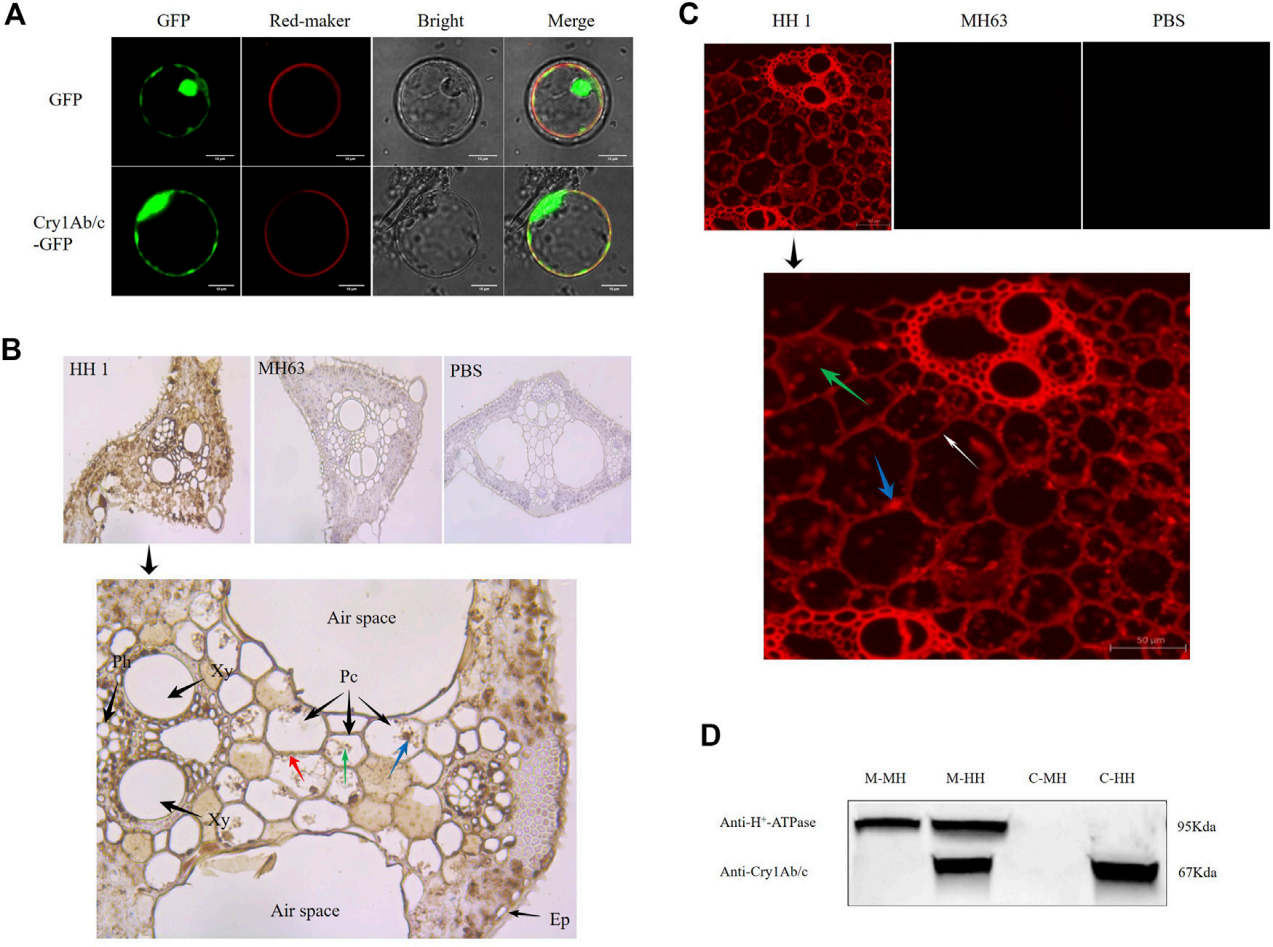
Cellular distribution of exogenous Cry1Ab/c protein in rice plant cells. **(A)**, Coexpression of Cry1Ab/c-GFP and red membrane-marker in rice protoplasts (scale bar: 10 μm). Coexpression of empty vector GFP and red membrane-marker was used as a negative control. **(B)**, Immunohistochemical analysis of exogenous Cry1Ab/c protein in mesophyll cells of transgenic rice HH1 (Cross sections of rice leaf), as indicated by the presence of brown-yellow particles (DAB staining). Ep, epidermis; Pc, Parenchyma cell; Xy, Xylem; Ph, Phloem. blue arrows indicate nuclei (hematoxylin), green arrows indicate cytoplasm, and red arrows indicate plasma membranes. MH63 rice and PBS were used as negative controls for protein expression and staining, respectively. **(C)**, Immunofluorescence analysis of exogenous Cry1Ab/c protein in mesophyll cells of transgenic rice HH1 (cross sections of rice leaf), as indicated by the red fluorescent signal. Leaf samples were incubated with an anti-Cry1Ab/c monoclonal antibody and a secondary antibody conjugated to the red-fluorescent Alexa Fluor 594 dye. White arrows indicate plasma membranes, green arrows indicate cytoplasm, and blue arrows indicate nuclear material. MH63 rice and PBS were used as negative controls, respectively. **(D)**, Western blot analysis of Cry1Ab/c protein in the plasma membrane and cytoplasm of rice plants. Membrane and cytosolic proteins (20 μg) from leaves of HH1 and MH63 were subjected for western blotting. C-MH, cytosolic protein fraction in MH63; C-HH, cytosolic protein fraction in HH1; M-MH, membrane protein fraction in MH63; M-HH, membrane protein fraction in HH. H^+^-ATPase was included in the analysis as a membrane indicator (about 95 KDa). The assay was repeated three times.

To visualize the subcellular localization of the Cry1Ab/c protein in plants, we transiently co-expressed Cry1Ab/c-GFP with a membrane marker in rice protoplasts. The Cry1Ab/c-GFP fusion protein was predominantly distributed on the cell membrane and in the cytoplasm and nucleus in rice protoplasts, as indicated by green fluorescent protein GFP (Figure 1A). Additionally, we transiently expressed a Cry1Ab/c-mCherry fusion protein in *Nicotiana benthamiana* mesophyll cells. The Cry1Ab/c-mCherry fusion protein was predominantly distributed on the cell membrane and in the cytoplasm and nucleus in *N. benthamiana* mesophyll cells, as indicated by mCherry red fluorescence signals (Supplementary Figure S1).

Next, the cellular distribution of Cry1Ab/c in HH1 and MH63 rice leaves was investigated through immunohistochemical analysis (Figure 1B). Nuclear and cytoplasmic Cry1Ab/c localization could not be observed in all cells, because plant cells are relatively large and contain large vacuoles. Hematoxylin is used to visualize nuclear contours. By observing the distribution of brown-yellow particles (DAB staining) in cells, we could determine that exogenous Cry1Ab/c is mainly located in the nucleus (brown-yellow particles were distributed in the nucleus), the cytoplasm (brown-yellow particles were evenly distributed throughout the cytoplasm), and on the cell membrane (brown-yellow particles formed a thin layer on the surface of the cell membrane). MH63 rice and phosphate-buffered saline (PBS) were used as negative controls for protein expression and staining, respectively, in which, as expected, no brown-yellow particles were observed.

In addition, we utilized *in-situ* immunofluorescence to visualize Cry1Ab/c distribution in rice leaves using an anti-Cry1Ab/c monoclonal antibody labeled with a secondary antibody conjugated to a red fluorescent dye (Figure 1C). In transgenic rice HH1, strong red fluorescent signals from Cry1Ab/c were observed on the cell membrane (which formed a thin layer on the surface of the cell membrane) and in the cytoplasm and nucleus. As expected, no signals were observed in the parental line MH63. Furthermore, control sections of HH1 leaves incubated with PBS were devoid of labeling. These results were consistent with the above-mentioned immunohistochemistry findings. We did not conduct nuclear staining in this experiment because of strong autofluorescence from the nuclear marker DAPI in plant leaves.

Finally, to further determine the plasma membrane distribution of Cry1Ab/c, which lacks an transmembrane domain through TMHMM prediction (Supplementary Figure S1), we separated membrane and cytosolic proteins from the leaves of HH1 and MH63. H^+^-ATPase, which was included in the analysis as a membrane indicator, was detected only in the plasma membrane protein fraction, not in the cytoplasmic protein fraction. Cry1Ab/c was detected in both the membrane and cytoplasmic fractions of transgenic rice HH1, but not in the parental line MH63 (Figure 1D and Supplementary Figure S4), indicating that exogenous Cry1Ab/c was located on the plasma membrane and in the cytoplasm. Therefore, Cry1Ab/c protein expression on the plasma membrane may affect the growth and development of HH1 rice.

### Screening of Cry1Ab/c-Interacting Endogenous Proteins

**FIGURE 2:**
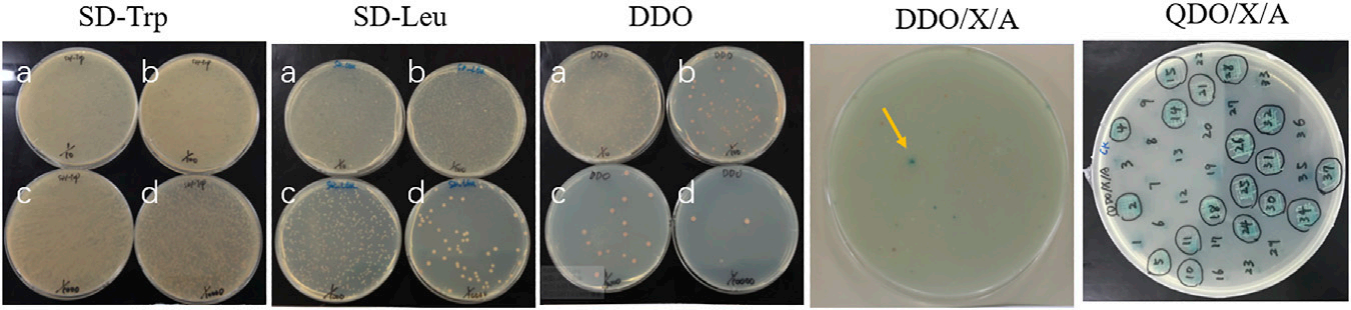
Cry1Ab/c-interacting endogenous proteins. The growth of co-transformed Y2H Gold yeast colonies harboring Cry1Ab/c-BD and cDNA-AD at nutrient-deficient SD/-Trp, SD-Leu, DDO, DDO/X/A and QDO/X/A, respectively. **(a–d)** The dilution ratio is 1/10, 1/100, 1/1000, 1/10,000, respectively. (BD) PGBKT7, (AD) PGADT7. Yellow arrow and black circle indicate positive clones.

To explore the possible reason for the plasma membrane localization of Cry1Ab/c, we conducted a large-scale functional screening of the rice endogenous proteins interacting with exogenous Cry1Ab/c, using a yeast two-hybrid (Y2H) screening. Eighteen positive clones could grow normally on quadruple nutrient-deficient SD/–Ade/–His/–Leu/–Trp/X/A (QDO/X/A) medium (Figure 2). After removing redundant gene sequences, 15 interactive proteins were identified, including the vacuolar membrane V-H^+^-ATPase and plasma membrane Ca^2+^-ATPase (Table 1). Ca^2+^-ATPase was transiently expressed in rice protoplasts to further clarify its localization on the plasma membrane, while V-H^+^-ATPase was not (Supplementary Figure S3). The potential interaction of plasma membrane Ca^2+^-ATPase and Cry1Ab/c may explain why the latter is largely located on the plasma membrane. However, the interaction between exogenous Cry1Ab/c and plasma membrane Ca^2+^-ATPase remains to be confirmed.

**TABLE 1:** Detailed gene names and accession numbers of positive clones that interact with Cry1Ab/c protein.

Name of protein interacting with Cry1Ab/c	Accession number in NCBI
Chromatin structure-remodeling complex protein SYD isoform X2	XM015786971.1
Patellin-3	XM015784751.1
Proline synthase co-transcribed bacterial homolog protein isoform X2	XM015777991.1
Protein SGT1 homolog	XM015766221.1
Proliferating cell nuclear antigen	XM015771759.1
HSP70	L32165.1
Bifunctional nuclease 1	XM015765834.1
AP-1 complex subunit mu-2	XM015782448.1
Putative wall-associated receptor kinase-like 16	XM015778712.1
Peptidyl-prolyl cis-trans isomerase FKBP12	XM015769882.1
ERBB-3 BINDING PROTEIN 1	XM015785210.1
Protein TSS	XP 015646988.1
Protein AE7-like 1	XM015772391.1
**Calcium-transporting ATPase 8, plasma membrane-type (Ca** ^**2+**^ **-ATPase)**	**XM015794313.2**
**V-type proton ATPase subunit E-like (V-H** ^**+**^ **-ATPase)**	**XM_015766313.1**

The bold black font indicates the candidate endogenous cell membrane protein.

### Interaction Between Exogenous Cry1Ab/c and Plasma Membrane Ca^2+^-ATPase

**FIGURE 3:**
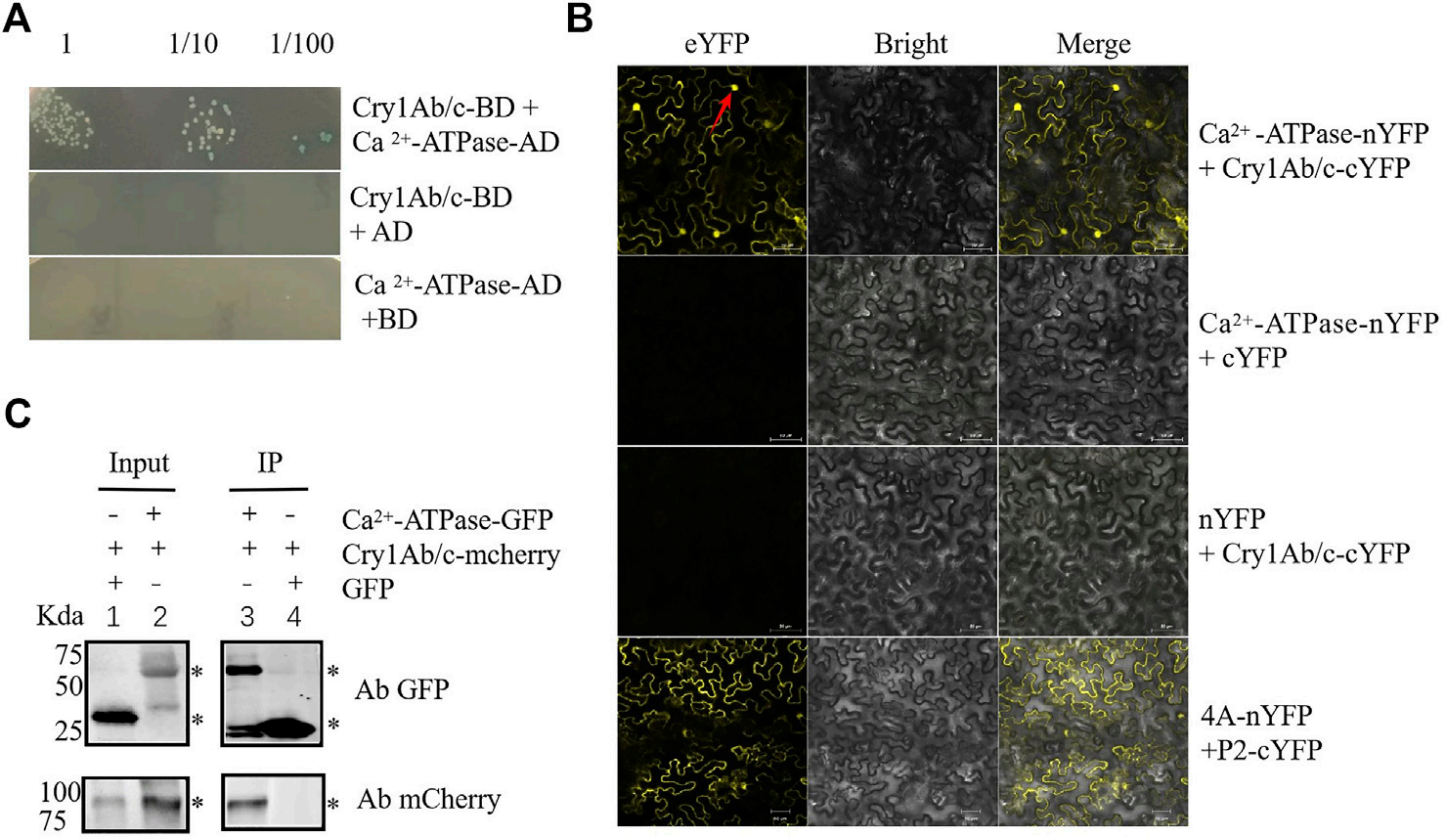
Cry1Ab/c interacts with plasma membrane Ca^2+^-ATPase. **(A)**, Interaction between Cry1Ab/c-AD and Ca^2+^-ATPase-BD in Y2H Gold yeast cells was verified through yeast two-hybrid experiments. Y2H Gold yeast cells co-transformed with the indicated plasmids were spotted on quadruple dropout medium with X-α-gal (SD/–Ade/–His/–Leu/–Trp/X/A). Empty pGBKT7 (BD) and pGADT7 (AD) vectors were used as negative controls. **(B)**, Interaction between exogenous Cry1Ab/c-cYFP and Ca^2+^-ATPase-nYFP fusion proteins in *N. benthamiana* mesophyll cells was verified through BiFC. YFP yellow fluorescence was observed upon complementation of the Cry1Ab/c-cYFP and Ca^2+^-ATPase-nYFP fusion proteins. cYFP coexpressed with Ca^2+^-ATPase-nYFP and nYFP coexpressed with Cry1Ab/c-cYFP were used as negative controls. 4A (NbeIF4A) plus P2 (RSV-encoded proteins) was used as the positive control. Red arrow indicates cell nuclei. scale bars: 50 μm **(C)**, Interaction between exogenous Cry1Ab/c-mCherry and Ca^2+^-ATPase-GFP fusion proteins in *N. benthamiana* mesophyll cells was verified through co-IP. Protein extracts (Input) were immunoprecipitated with GFP-trap agarose beads (IP) and resolved by SDS-PAGE. The immunoblots shown were developed with anti-GFP antibody to detect Ca^2+^-ATPase-GFP fusion protein (54 kDa) and with anti-mCherry antibody to detect Cry1Ab/c-mCherry fusion protein (94 kDa). GFP empty vector plus Cry1Ab/c-mCherry was used as a negative control (27 kDa). “*” indicates the target band. The assays were repeated three times.

To verify the Cry1Ab/c–Ca^2+^-ATPase interaction, we first used one-on-one Y2H. The Y2H Gold yeast strains co-transformed with Cry1Ab/c-AD and Ca^2+^-ATPase-BD could grow normally on quadruple dropout media with X-α-gal, whereas yeast cells carrying two control constructs could not. These results indicated that exogenous Cry1Ab/c can interact with plasma membrane Ca^2+^-ATPase in yeast cells (Figure 3A). Next, BiFC was used to confirm the protein interaction in planta. Protein interaction sites and strength were detected by *in-situ* yellow fluorescent protein (YFP) fluorescence analysis. Exogenous Cry1Ab/c-nYFP strongly interacted with endogenous Ca^2+^-ATPase-cYFP in tobacco mesophyll cells, and the interaction produced YFP signals on the plasma membrane and nucleus, whereas no YFP signal was observed in negative control cells harboring an empty vector (Figure 3B). Co-immunoprecipitation (Co-IP) reflects protein interactions in the natural state in living plant cells. It helps eliminate false-positives from BiFC caused by spontaneous fluorescence. A Cry1Ab/c-mCherry fusion protein could be pulled down with Ca^2+^-ATPase-GFP, whereas an empty vector harboring GFP could not (Figure 3C and Supplementary Figure S5). Taken together, these results consistently demonstrated that Cry1Ab/c-mCherry could interact with plasma membrane Ca^2+^-ATPase-GFP *in vitro*.

### Determination of Phenotypic Indices and Ca^2+^-ATPase Activity in Rice Lines Under Salt Stress

As Ca^2+^-ATPase-GFP was found to be distributed on the plasma membrane, whereas its interaction sites with Cry1Ab/c were distributed on the plasma membrane and in the nucleus, we speculated that Cry1Ab/c protein expression might retain some Ca^2+^-ATPase-GFP protein in the nucleus and thus affect Ca^2+^-ATPase activity on the membrane.

**FIGURE 4:**
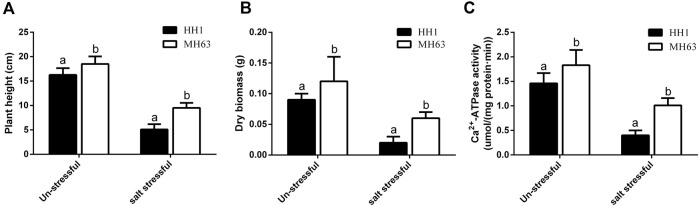
Determination of phenotypic indices and Ca^2+^-ATPase activity in rice plants under stress-free and salt-stress conditions. **(A)**, Plant heights of HH1 and MH63 rice plants under stress-free and salt-stress conditions. **(B)**, Dry biomass of HH1 and MH63 rice plants under stress-free and salt-stress conditions. **(C)**, Ca^2+^-ATPase activity in HH1 and MH63 rice plants under stress-free and salt-stress conditions. Lowercase letters indicate significant differences among treatment means (*p* ≤ 0.05; Student’s *t*-test).

To confirm this hypothesis, phenotypic indices and Ca^2+^-ATPase activity in HH1 and MH63 were determined under stress-free and salt-stress conditions. Plant height and dry biomass were significantly higher under stress-free conditions than under salt stress in both lines, but significantly lower in HH1 rice than in MH63 rice under both conditions (Figures 4A,B). Ca^2+^-ATPase activity was significantly lower under salt stress than under stress-free conditions, but significantly lower in HH1 rice than in MH63 rice under both conditions (Figure 4C). Overall, the differences in these indices and Ca^2+^-ATPase activity was relatively higher under salt-stress condition than that of the rice under farmland soil condition. These results indicated that a specific interaction between Cry1Ab/c and the plasma membrane protein Ca^2+^-ATPase may affect the Ca^2+^-ATPase activity on the plasma membrane of HH1, thereby affecting the salt resistance of HH1.

## Discussion

There is a wealth of information on unintended effects in transgenic crops; nevertheless, little is known about the underlying mechanisms. We discovered that the exogenous Cry1Ab/c protein has an affinity for the plant cell plasma membrane, which likely is related to its interaction with the endogenous plasma membrane Ca^2+^-ATPase. Seemingly, this interaction negatively regulates salt-stress resistance in transgenic plants by decreasing the Ca^2+^-ATPase activity on the membrane.

The cellular localization of exogenous proteins in plants not only determines the stability of their expression but also plays an important role in their effects on plant growth and development. We showed that exogenous Cry1Ab/c protein was located not only in the nucleus and cytoplasm but also on the plasma membrane in HH1 transgenic rice. Similarly, Zhang (1999) found that exogenous Bt protein is lowly expressed in the cytoplasm and highly expressed in the plasma membrane and speculated that it may bind to the plasma membrane. Dong et al. (2006) used *in-situ* immunohistochemistry to determine that Cry1Ab/c was mainly distributed in the cytoplasm, plasma membrane, and intercellular spaces in insect-resistant transgenic cotton. Liu et al. (2011) reported that a transiently expressed Cry1Ac22-eGFP fusion protein produced strong fluorescence in the yeast plasma membrane, indicating that Cry1Ac22 is located in the yeast plasma membrane. The current and previous findings suggest that exogenous Cry1Ab/c protein may have an affinity for the plasma membrane. To our knowledge, Cry toxins are receptor-binding, oligomerizing, and pore-forming proteins (Gómez et al., 2006; Pacheco et al., 2009; Adang et al., 2014); thus, the plasma membrane localization of Cry1Ab/c might hinder normal physiological processes. For example, it may explain our previous finding that vegetative and reproductive indices, such as tiller number, biomass, and yield per plant, were significantly lower in HH1 than in MH63 under farmland soil conditions (Fu et al., 2018). However, plasma membrane damage caused by Cry1Ab/c protein expression remains to be confirmed using artificial membranes.

Considering that Cry1Ab/c does not have an obvious transmembrane domain, the fact that it is localized largely on the plasma membrane and has an affinity for the plasma membrane was worth exploring. Thus, we conducted large-scale Y2H screening and identification of rice endogenous proteins that interact with Cry1Ab/c. Interestingly, we used Y2H, BiFC, and co-IP assays to determine that the plasma membrane Ca^2+^-ATPase can interact with Cry1Ab/c. We speculated that Cry1Ab/c accumulates on the plasma membrane *via* its interaction with endogenous plasma membrane Ca^2+^-ATPase.

Ca^2+^-ATPase, a key player in transmembrane transport and signal transduction, forms complex transmembrane signaling pathways in response to external stresses (Luoni et al., 2006; Qudeimat et al., 2009). Therefore, interactions of Ca^2+^-ATPase with other proteins may influence stress resistance in plants. We found that the cellular localization of plasma membrane protein Ca^2+^-ATPase and its site of interaction with Cry1Ab/c were different by a BIFC experiment, which may affect Ca^2+^-ATPase activity on the membrane by partially retain it in the nucleus. Similar a study showed that 14-3-3 protein interacts with the leucine zipper structure transcription factor RSG in tobacco, which causes the latter to be retained in the cytoplasm to negatively regulate the gibberellin signaling pathway, and thus affecting the adaptability of plants to adversity (Ishida et al., 2004). Purwestri et al. (2017) found that the 14-3-3 protein GF14e could interact with Hd3a, and thus leading to Hd3a cyoplasmic retention; the increasing of cytoplasmic retention of Hd3a can be explained as a model of late flowering by overexpression of GF14e. Therefore, the change in the subcellular localization of target proteins maybe very important for affecting their function in a cell. However, we can not ignore that deeply understanding the interaction mechanism between Cry1Ab/c and plasma membrane protein Ca^2+^-ATPase would seem to go beyond location. In the future, we will include some aspects such as molecular orientation, binding, inhibition or stimulation and so on to deeply confirm this interaction mechanism.

Actually, our further experiments showed that, under salt stress, Ca^2+^-ATPase activity was significantly lower in HH1 rice than in MH63 rice; additionally, plant height and biomass were also significantly lower than those in MH63 rice, which indicated that HH1 has lower salt tolerance than MH63. These findings support that the Cry1Ab/c–Ca^2+^-ATPase interaction may negatively regulate plant salt-stress resistance. Astegno et al. (2017) found that the Ca^2+^ sensor CML36 directly interacted with *Arabidopsis* plasma membrane Ca^2+^-ATPase isoform 8 (ACA8) and that this interaction affected Ca^2+^-ATPase activity. Another similar study confirmed that ACA8 and CIPK9 (calcineurin B-like protein-interacting protein kinase) interaction affects ACA8 activity, thus provoking relevant functional consequence, including shapes the cytosolic Ca^2+^ transients induced by mechanical wounding of leaves (Costa et al., 2017). The height of the second peak of the wound-induced Ca^2+^ transient. Further studies are needed to determine how the Cry1Ab/c–Ca^2+^-ATPase interaction affects Ca^2+^-ATPase activity in HH1 plants under salt stress, thereby causing a series of changes in their regulated pathways and finally producing discernible pleiotropic effects of the phenotypes. And further experiments needs to prove the effect of Ca^2+^-ATPase on the growth and development of transgenic rice plants and salt tolerance traits through overexpression and CRISPR/Cas9 gene editing technology of Ca^2+^-ATPase. However, we can not ignore that genetic background maybe another reason for explaining these significant unintended effects (Wang et al., 2016).

## Conclusion

To the best of our knowledge, this is the first study to elucidate the molecular mechanism underlying the unintended effects of protein interaction in transgenic crops, and to identify Ca^2+^-ATPase as an endogenous target protein of exogenous Cry1Ab/c. We argue that the interaction of Ca^2+^-ATPase and Cry1Ab/c may not only explain the plasma membrane location of Cry1Ab/c but also affects the salt resistance of HH1, likely by decreasing Ca^2+^-ATPase activity under salt stress; however, more evidence is required to confirm this hypothesis. These findings provide a sound theoretical basis for elucidating the mechanism of its adverse effects on plant growth and development. These findings can also be used to ensure food security, maintain or enhance crop quality and yield, and increase profitability for the grower and the local agroeconomy.

## Author's Note

The present study revealed that exogenous insecticidal toxin Cry1Ab/c from *Bacillus thuringiensis* expressed in transgenic rice Huahui-1 binds the plasma membrane Ca^2+^-ATPase to negatively regulate salt resistance in Huahui-1, likely by decreasing Ca^2+^-ATPase activity under salt stress.

## Data Availability

The original contributions presented in the study are included in the article/Supplementary Material, further inquiries can be directed to the corresponding author.

## Abbreviations

HH1, Huahui-1; MH63, Minghui63; ORF, open reading frame; NCBI, National Center for Biotechnology Information; nYFP, PCV-cYFP-N; cYFP, PCV-nYFP-C; BD, pGBKT7; AD, pGADT7; mCherry, pBINPLUS-mCherry; GFP, pBINPLUS-GFP; YFP, Yellow fluorescence protein; SDS-PAGE, Sodium dodecyl sulfate polyacrylamide gel electrophoresis; ECL, Electrochemiluminescence; DDO, SD-Trp-Leu; QDO, SD-Trp-Leu-His-Ade; X, X-α-gal; A, Aureobasidin A; NCBI, National Center for Biotechnology Information; Bt, *Bacillus thuringiensis*; Y2H, yeast two-hybrid; BIFC, Bimolecular fluorescence complementation (BIFC); Co-IP, Co-immunoprecipitation; GFP, Green fluorescent protein; PBS, phosphate-buffered saline; DAB, 3,3′-Diaminobenzidine tetrahydrochloride
