# Supplementary material for: Cellular Localization of Exogenous Cry1Ab/c and its Interaction with Plasma Membrane Ca2+-ATPase in Transgenic Rice
Source: Front Bioeng Biotechnol. 2021 Nov 2;9:759016. doi: 10.3389/fbioe.2021.759016 (PMC8596563; doi:10.3389/fbioe.2021.759016)
Supplement: Supplementary file 1 [file DataSheet1.docx]

**Supplementary information**


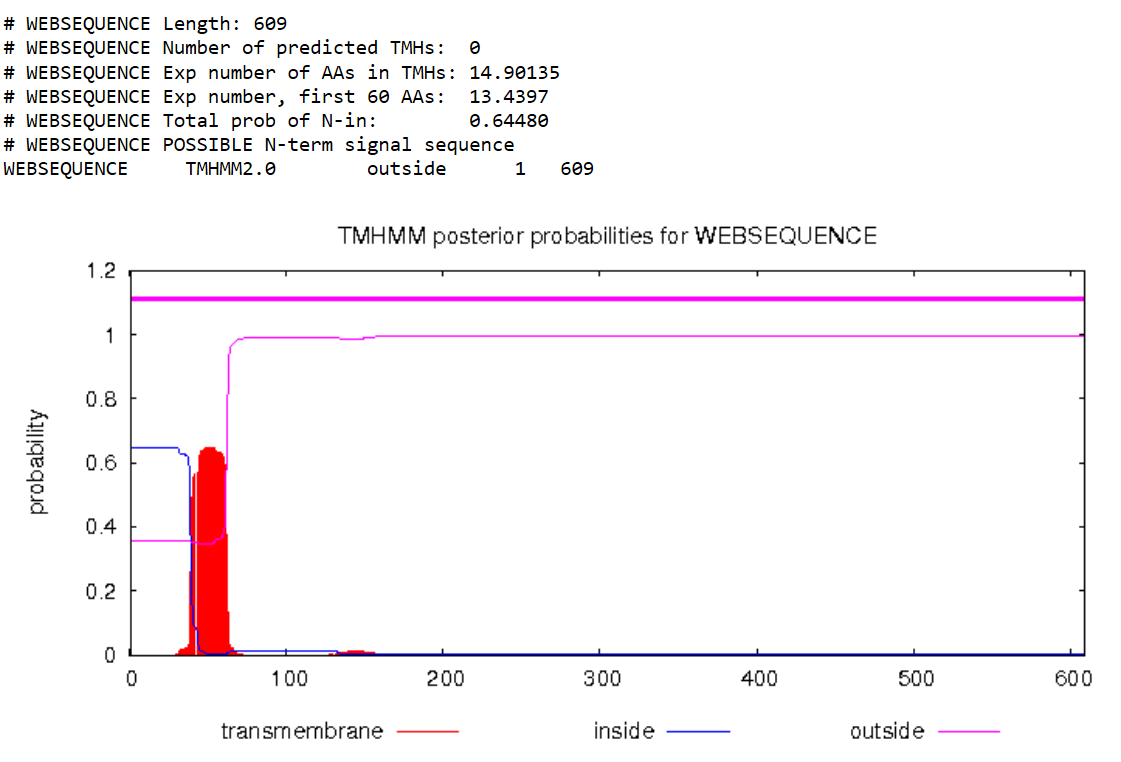


**FIGURE S1 The transmembrane domain of Cry1Ab/c protein was predicted by TMHMM online software.**


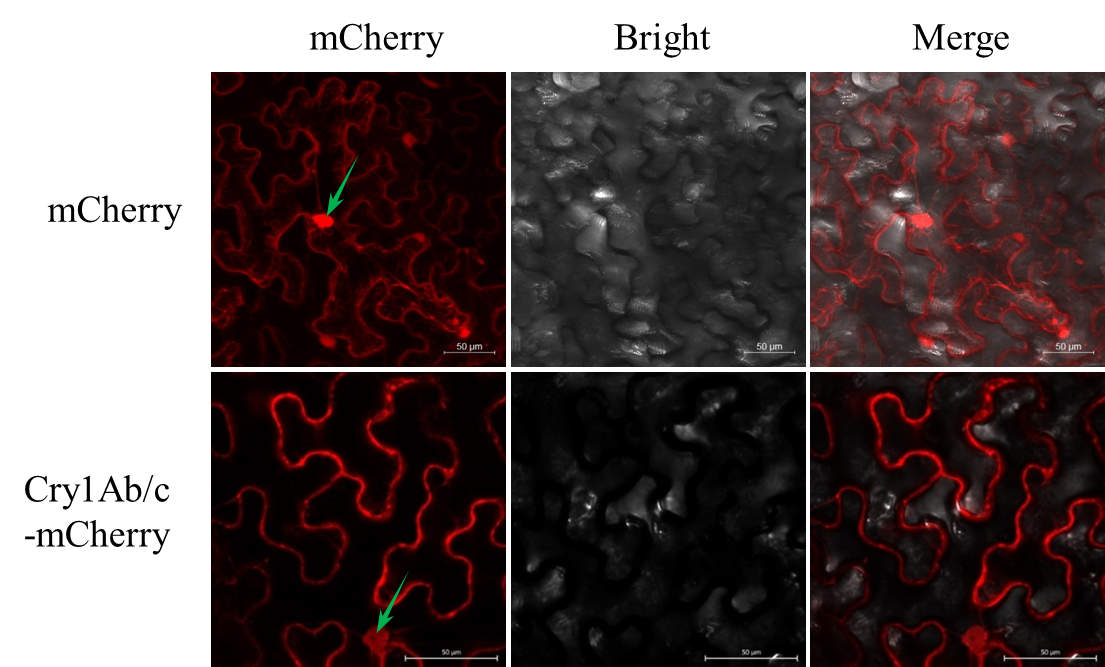


**FIGURE S2 Subcellular localization of exogenous Cry1Ab/c protein in *N. benthamiana* mesophyll cells (scale bar: 50 μm).** Empty mCherry vector was used as a negative control. Blue arrows indicate nuclear.


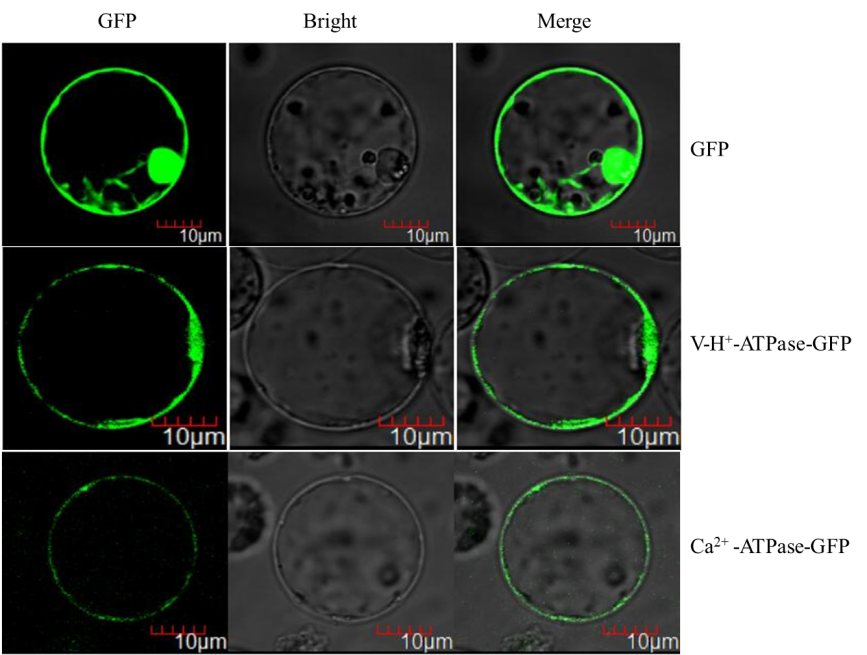


**FIGURE S3 Subcellular localization of V-H^+^-ATPase-GFP and Ca^2+^-ATPase-GFP in rice protoplast**. GFP empty vector was used as a control, Scale bars: 10μm.


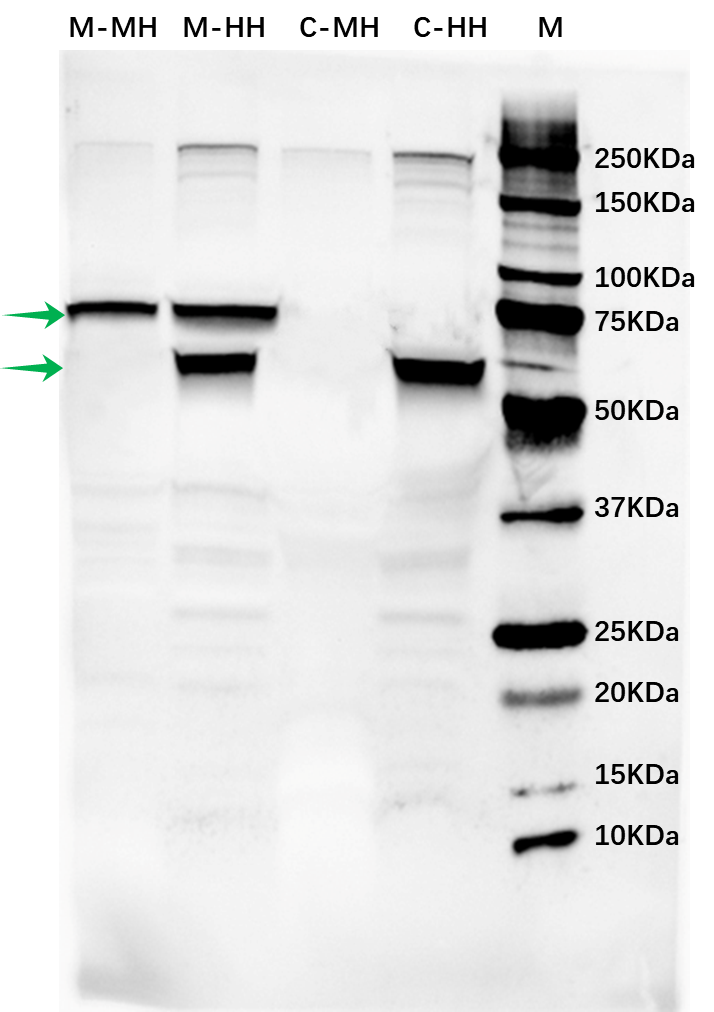


**FIGURE S4 Quantitative analysis of Cry1Ab/c protein expression in the plasma membrane and cytoplasm of rice plants (Original images)**. Membrane and cytosolic proteins from leaves of HH1 and MH63 were subjected to western blotting. a, Cry1Ab/c protein accumulates on the plasma membrane and in the cytoplasm. Equal amounts of protein (20 μg) were loaded in each lane. C-MH, cytosolic protein fraction in MH63; C-HH, cytosolic protein fraction in HH1; M-MH, membrane protein fraction in MH63; M-HH, membrane protein fraction in HH. H+-ATPase was included in the analysis as a membrane indicator (about 95KDa).

**
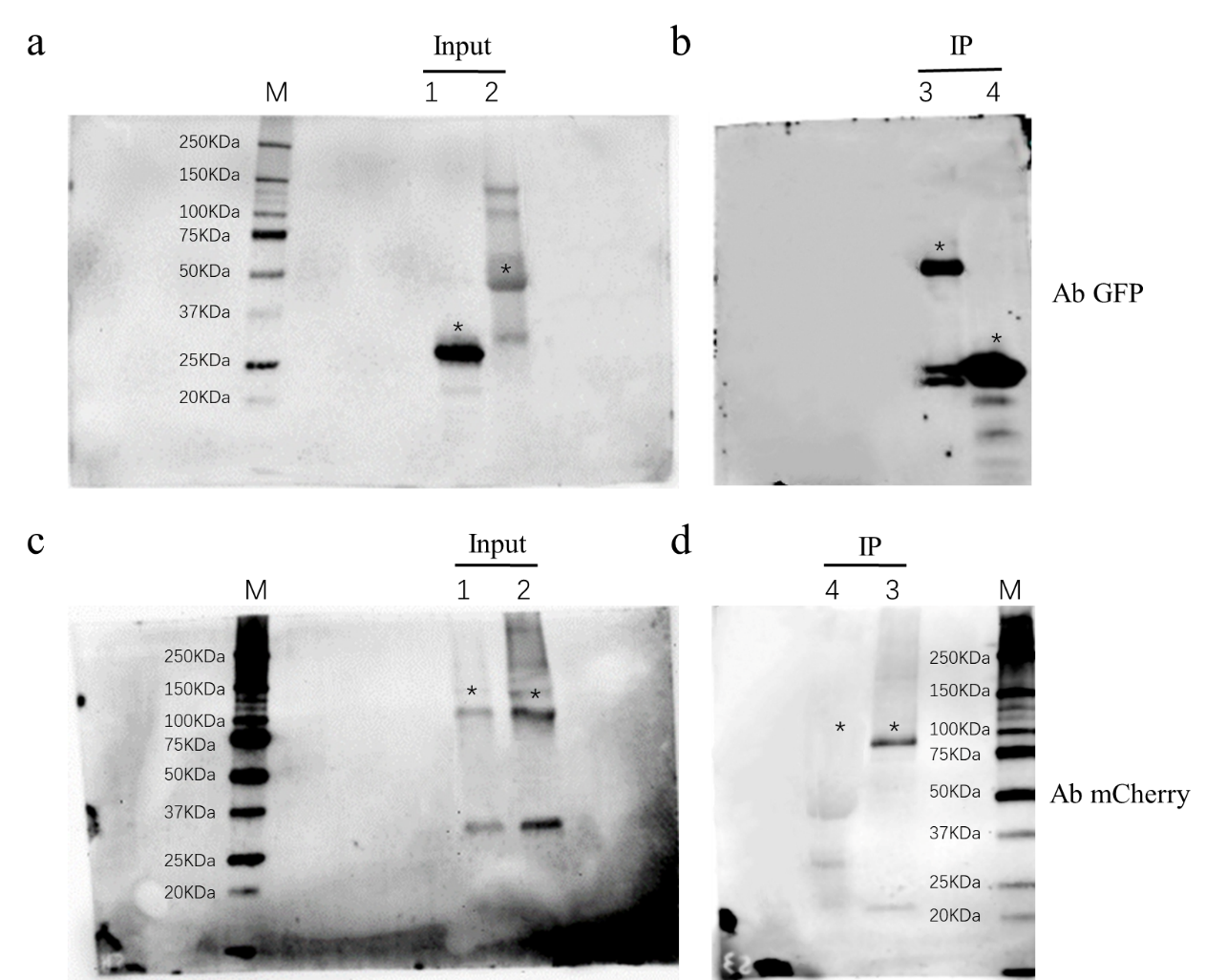
**

**FIGURE S5 Interaction between exogenous Cry1Ab/c-mCherry and Ca^2+^-ATPase-GFP fusion proteins in *N. benthamiana* mesophyll cells was verified through co-IP (Original images)**. Protein extracts (Input) were immunoprecipitated with GFP-trap agarose beads (IP) and resolved by SDS-PAGE. The immunoblots shown were developed with anti-GFP antibody to detect Ca^2+^-ATPase-GFP fusion protein (52kDa) and with anti-mCherry antibody to detect Cry1Ab/c-mCherry fusion protein (94 kDa). GFP empty vector plus Cry1Ab/c-mCherry was used as a negative control (27 kDa). (a,c) GFP + Cry1Ab/c-cherry (27 kDa, lanes 1 and 4); (b,d) Ca^2+^-ATPase-GFP + Cry1Ab/c-cherry (47 kDa, lanes 2 and 3). “*” indicates the target band. The assays were repeated three times.

**Table S1** Primers for cloning the full length gene based on subcellular co-localization and co- immunoprecipitation vectors

| Primer name | Sequences |
| --- | --- |
| Ca^2+^-ATPase-BD -F | CATGGAGGCCGAATTCATGGCGCTCGGGAGGTC |
| Ca^2+^-ATPase-BD -R | GGATCCCCGGGAATTCTTAGCTCTGTCCACTCTCCTCTTCA |
| Cry1Ab/c-AD-F | GGAGGCCAGTGAATTCATGGACAACTGCAGGCCATAC |
| Cry1Ab/c-AD-R | CACCCGGGTGGAATTCTTATTCAGCCTCGAGTGTTGC |
| Ca^2+^-ATPase-nYFP -F | CGGGAGATGCGGATCCATGGCGTCCACCTCCTGCT |
| Ca^2+^-ATPase-nYFP-R | GCTCGCCTGGGGATCCTGCGACGCTGAAGGAGCTGGCTGCG |
| Cry1Ab/c- cYFP-F | CGGGAGATGCGGATCCATGGACAACTGCAGGCCATAC |
| Cry1Ab/c- cYFP-R | GCTCGCCTGGGGATCCTTATTCAGCCTCGAGTGTTGC |
| V-H^+^-ATPase-GFP4-F | CGGGGTCGACGGATCCATGAACGACGCCGATGTCG |
| V-H^+^-ATPase-GFP4-R | TGCTCACCATGGATCCTGCCGTCACCTGACCAA |
| Ca^2+^-ATPase-GFP4-F | CGGGGTCGACGGATCCATGGCGCTCGGGAGGTC |
| Ca^2+^-ATPase-GFP4-R | TGCTCACCATGGATCCGCTCTGTCCACTCTCCTCTTCA |
| Cry1Ab/c-mCherry-F | CGGGGTCGACGGATCCATGGACAACTGCAGGCCATAC |
| Cry1Ab/c-mCherry-R | TGCTCACCATGGATCCTTCAGCCTCGAGTGTTGC |
